# Morphometric Analysis of the Tibial Tunnel after Primary and Revision Anterior Cruciate Ligament Reconstruction

**DOI:** 10.5704/MOJ.2503.009

**Published:** 2025-03

**Authors:** E Veizi, N Cay, BS Sezgin, A Sahin, A Firat, M Bozkurt

**Affiliations:** 1 Department of Orthopaedics and Traumatology, Ankara Bilkent City Hospital, Ankara, Turkey; 2 Department of Radiology, Ankara Bilkent City Hospital, Ankara, Turkey; 3 Department of Orthopaedics and Traumatology, Ankara Acibadem Hospital, Ankara, Turkey; 4 Department of Orthopaedics and Traumatology, VM Medicalpark Ankara Hospital, Ankara, Turkey

**Keywords:** morphometric measurements, anterior cruciate ligament, tibial tunnel, tibial tuberosity

## Abstract

**Introduction:**

Anterior cruciate ligament (ACL) reconstruction is a commonly performed surgical procedure. The objectives of this retrospective comparative study are (1) to evaluate the obliquity, size and the intra-articular aperture shape of the tibial tunnel in patients operated with an anteromedial portal technique, and (2) to determine their possible relation with revision surgery.

**Material and Methods:**

Patients operated for a primary ACL reconstruction between 2014 and 2018 were eligible. All patients of primary and revision ACL fulfilling the inclusion criteria were assessed for presence of a knee CT scan within one month of surgery and at least three years of follow-up. Several radiological parameters were measured for the study, among which: Tunnel height, Coronal tunnel angle, Maximal tunnel width and Sagittal tunnel inclination. Multivariate analyses were performed to identify parameters correlated with revision.

**Results:**

Mean age of the primary group was 30.5±8.4 versus 29.4±8.0 of the revision group. The majority of patients were males in both groups (n=33, 76.7% and n=38, 95.0%, respectively). A longer diameter of the intra-articular ellipse (p=0.005) and an increased mid-tunnel to TT distance on the axial plane (p=0.006) were significantly correlated with revision. A ROC curve analysis determined a cut-off value of 27.9mm from the tubercle was an optimal entry point.

**Conclusion:**

A greater distance between the mid-point of the tibial tunnel entrance and the centre of the tibial tubercle is linked to a higher risk of revision. An elongated elliptic shape in the antero-posterior plane also correlates with revision risk.

## Introduction

Anterior cruciate ligament (ACL) reconstruction is the most commonly performed surgical procedure for a ruptured ligament in young active patients with good long-term results^1,2^. Especially in the last decade the concept and the surgery itself have been developed and refined while controversies regarding femoral and tibial tunnel placement are still ongoing^3-5^.

Usage of an additional anteromedial portal to create the femoral tunnel instead of the more traditional transtibial technique is currently the preferred approach for reconstruction, since it yields better rotational stability and restores antero-posterior translation during walking^4,6^. This technique also lacks the drawbacks of the transtibial one since the tibial tunnel is only reamed once during preparation and is not used as an aiming corridor for femoral preparation^6^. Miller *et al* showed that during a transtibial femoral site preparation, the tibial tunnel itself and its intra-articular aperture is enlarged, changing the obliquity of the tunnel and resulting in a tunnel-graft size mismatch^7^. Full graft accommodation on the intra-articular aperture is important to avoid greater synovial fluid from entering the tunnel^8,9^ with many authors having demonstrated higher graft stresses in such cases^10^.

Research on the tibial tunnel orientation and its entry/exit apertures have been greatly overshadowed in the past decade by the high volume of research focused on the femoral tunnel, its obliquity within the lateral condyle and its anatomical starting points^6,11^. Cadaveric and clinical studies have shown that usage of the anterior root of the lateral meniscus or the stump of the ruptured ACL usually led to optimal graft placements, but tibial tunnel malpositioning is still a frequent phenomenon^12^. While the functional relevance of this ‘malpositioning’ is still debatable, even fewer studies have focused on the entry point of the tibial tunnel at the level of the medial tibial metaphysis. The aim of this study is to evaluate the obliquity, size and the intra-articular aperture shape of the tibial tunnel in patients operated with an anteromedial portal technique and to determine their possible relation with revision surgery.

## Materials and Methods

For patient selection, the ethics committee of our institution (Ethics Committee Nr.2 - no. E2-22-1544, date 16.03.2022) reviewed and approved the design of the present retrospective study. We evaluated two groups of patients; those who just underwent a primary ACL reconstruction and those who were about to undergo revision surgery. Patients operated for an ACL rupture and treated with a primary reconstruction through an anteromedial portal technique between January 2014 and January 2018 at our institution were eligible for the first group of this study. Inclusion criteria were (1) patients over the age of 18, (2) an acute or chronic isolated ACL rupture diagnosis, (3) reconstruction performed with a hamstring tendon autograft, (4) patients who were at least on their 3rd post-operative year and (5) patients who underwent a knee CT scan within one month of the designated surgery. Exclusion criteria were revision at any time, prior ipsilateral knee surgery, additional ligamentous injury/surgery, usage of a transtibial technique or an autograft other than the hamstring and refusal to participate in this study.

Fifty-one patients met the inclusion criteria for the primary group. Demographical data was obtained from the medical records and follow-up clinical data was gathered prospectively during follow-up visits. A low-dose CT scan was a routinely performed procedure during those years at our institution and it was generally performed during the first post-operative month.

**Fig. 1:**
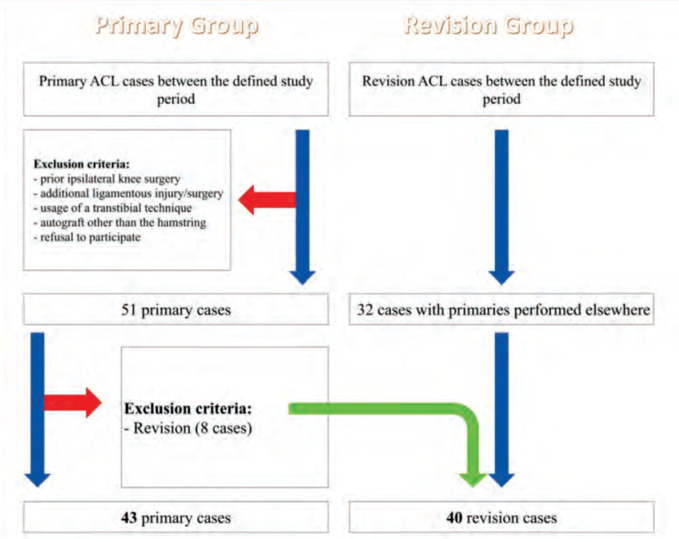
Flowchart of the included study groups.

In order to compare morphometric parameters, an independent cohort of only revision surgeries, operated between the same time frame, was also evaluated for this study. Routine CT scans are taken before revision surgeries and the cases were investigated for confounding factors; femoral tunnel malposition, hardware failure and slope >12°. After applying the aforementioned parameters, 40 revision cases were included into the study (Fig. 1). Their demographic and morphometric data was collected and used for comparison with the primary group.

For surgical technique and rehabilitation protocol, all surgeries were performed by the same team with the same attending surgeon either performing the procedures himself or supervising and directing the procedure closely. After a routine diagnostical arthroscopy, both hamstring tendon grafts were harvested in a standard fashion with a small incision on the pes anserinus^13^. While the grafts were being prepared for insertion, concomitant meniscal lesions were addressed. As a general rule during the study period, the graft was four-folded double-looped and its diameter was measured. Then the femoral tunnel was prepared from the anteromedial portal aiming for the native spot of the anteromedial band of the ACL over the resident ridge^14^.

The tibial guide [Smith and Nephew, Memphis, TN, USA], as a study rule, was always set at 55O and the stump of the ruptured ACL, just medial to the anterior horn of the lateral meniscus was the routinely aimed exit point. A passing pin of 2.4mm was firstly advanced and the entry point at the level of the medial proximal tibia was aimed midway between the posterior cortex of the proximal tibia and the medial margin of the tibial tuberosity. Care was taken not to injure the medial collateral ligament (MCL). The tunnel was then drilled in accordance with the graft’s thickness.

The autograft was then passed through the tunnels and fixed on the femoral side with an Endobutton® [Smith and Nephew, USA] and on the tibial side with a bioabsorbable interference screw and a ‘U’ staple. Again, as a study rule, the same technique was used for all cases. After proper irrigation the wound was closed in a standard fashion.

Patients followed the same standardised rehabilitation program. On the second post-operative day closed chain kinetic exercises were started while full weight bearing and strengthening exercises were allowed after the first week. Patients with concomitant meniscal repair were allowed to partially bear weight for six weeks. Full range of motion was gained at the 6th week after surgery. The patients were followed-up monthly for the first three months and then once every three months until the first post-operative year. Yearly routine visits were advised after that. Revision procedures were performed by more than one surgeon, with some of the cases being from our primary ones.

**Fig. 2:**
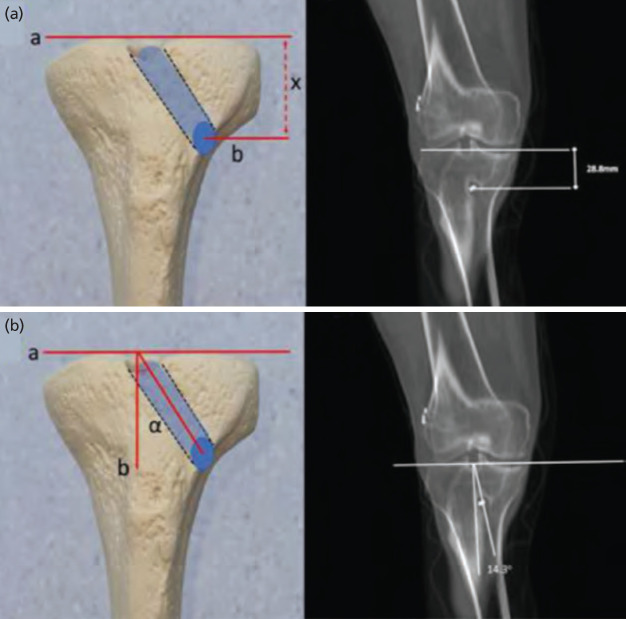
(a) The distance between the centre of the distal entry point and the joint line was measured and defined as ‘Tunnel height (1)’. (d) The centres of the entry and exit apertures were combined with a line and then the angle between this line and the line perpendicular to the joint line was measured and denoted as the ‘Coronal tunnel angle (2)’.

For radiological evaluations and measurements, the radiological imaging scans were performed with 128-slice CT [Revolution EVO, GE Healthcare Japan Corporation, Japan]. Patients were scanned in supine position. Axial 1.25mm slices were initially obtained and subsequently reconstructed for multiplanar view. On coronal images, the plane crossing the entry point of the tunnel on the tibial metaphysis and the plane where the exit point of the tunnel (intra-articularly) is best seen, were overlapped. In this overlapped image, the distance between the centre of the distal entry point and the joint line was measured and defined as ‘Tunnel height (1)’ (Fig. 2). Also on this image, the centres of the two (entry and exit) points were combined by drawing a line and then the angle between this line and the line perpendicular to the joint line was measured. This parameter was defined as ‘Coronal tunnel angle (2)’ (Fig. 2).

**Fig. 3:**
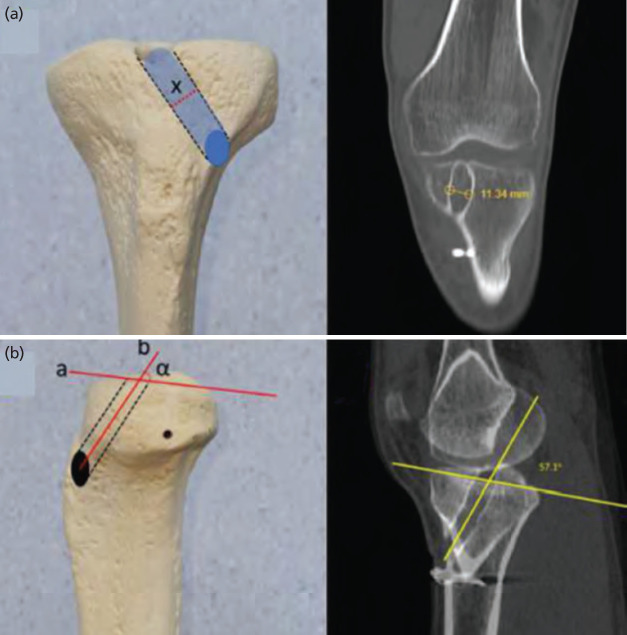
(a) On coronal images ‘Maximal tunnel width (3)’ was measured at the widest site of the tunnel. (b) On the sagittal plane, the inclination angle, between the sagittal mid-axis of the tunnel and the medial plateau surface was measured and denoted as ‘Sagittal tunnel inclination (4)’.

Again, on coronal images, slices were set manually parallel to tibial tunnels. On these reconstructed images ‘Maximal tunnel width (3)’ was measured at the widest site of the tunnel. On the sagittal plane, the inclination angle between the sagittal mid-axis of the tunnel and the medial plateau surface was measured and denoted as ‘Sagittal tunnel inclination (4)’ (Fig. 3).

**Fig. 4:**
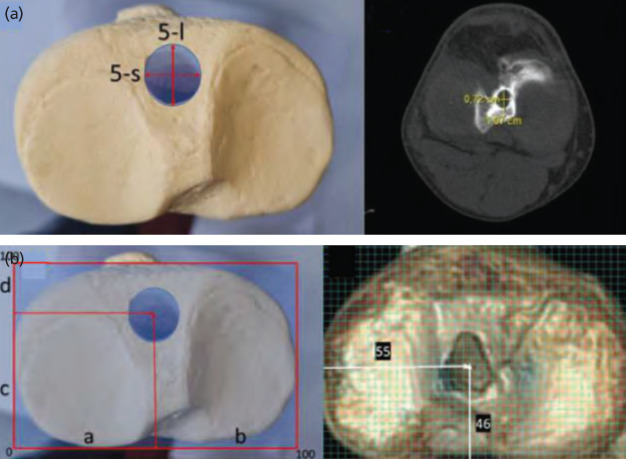
(a) On axial images, anterior-posterior and medial-lateral diameters of the exiting ellipsoid aperture was measured, and the parameters were defined as ‘Long diameter of exit ellipse (5-l)’ and ‘Short diameter of exit ellipse (5-s)’. (b) The centre of the tunnel aperture was identified with the grid method and the location was expressed as a percentage of tibial plateau anterior to posterior and medial to lateral depth using the parameters ‘Axial grid X position (6-x)’ and ‘Axial grid Y position (6-y)’.

On axial images, anterior-posterior and medial-lateral diameters of the exiting ellipsoid aperture of the tibial tunnel was measured and the parameters were defined as ‘Long diameter of exit ellipse (5-l)’ and ‘Short diameter of exit ellipse (5-s)’. The grid method^15^ was used to locate the mediolateral and anteroposterior location of the intra-articular exit point. The antero-posterior and medio-lateral bony edges of the tibia were identified, and a grid system was overlaid using ImageJ [National Institutes of Health, Bethesda, MD, USA]. The centre of the tunnel aperture was identified within the grid and the location was expressed as a percentage of tibial plateau anterior to posterior and medial to lateral depth. The parameters were identified as ‘Axial grid X position (6-x)’ and ‘Axial grid Y position (6-y)’ (Fig. 4).

**Fig. 5:**
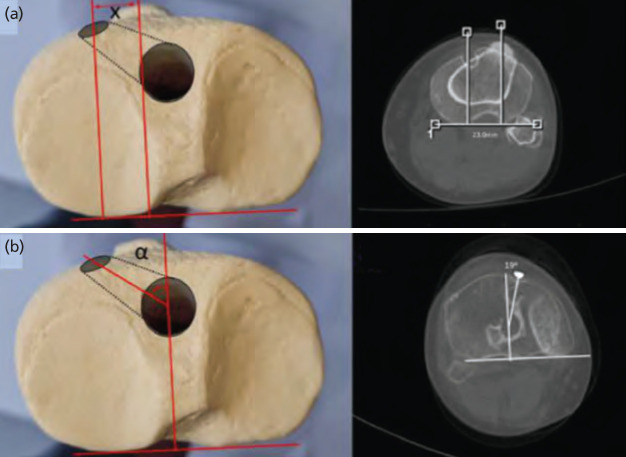
(a) On the axial plane, the distance between the tibial tunnel’s entry point and the mid-point of the tibial tuberosity was measured and denoted as ‘Axial mid-tunnel distance to TT (7)’. (b) The ‘Axial tibial tunnel index angle (8)’ was measured through a line perpendicular to the posterior condylar line and a line passing through the mid-point of the entry aperture on the tibial metaphysis. The images were overlapped, and an angle was obtained.

On the axial plane, a line tangent to the posterior tibial condyles was used as a reference to measure the distance between the tibial tunnel’s entry point and the mid-point of the tibial tuberosity. Two perpendicular lines, one passing through the mid-point of the tibial tuberosity and the other passing through the mid-point of the entry aperture were used to measure ‘Axial mid-tunnel distance to TT (7)’ parameter. The tibial tubercle was used as a reference because it is easily identifiable and is used intra-operatively to orientate the position of the tunnel. Finally, on the axial plane, the ‘Axial tibial tunnel index angle (8)’ was measured through a line perpendicular to the posterior condylar line and a line passing through the mid-point of the entry aperture on the tibial metaphysis. The images were overlapped, and an angle was obtained (Fig. 5). All measurements were performed by an experienced musculoskeletal radiologist, blinded to the study protocol, on two separate occasions and a week apart from each other.

For statistical analysis, the study data was analysed using SPSS 22.0 software [Chicago, IL, USA]. For descriptive analyses, categorical variables were stated as number (n) and percentage (%) while continuous variables were presented as mean ± standard deviation (SD) and median (minimum-maximum). Mann Whitney U-test was used for non-normally distributed data. Spearman and Pearson correlation tests were used to evaluate correlation between the study parameters. Revised cases were of primary interest therefore parameters being significantly correlated with revision were analysed with a univariate and a multivariate logistic regression model. A ROC analyses was performed for all parameters who were found to have a significant impact on revision in order to determine a cut-out value. Radiological measurements were performed twice by the same experienced musculoskeletal radiologist, on two separate occasions. Intra-observer reliability was analysed, and an intra-class correlation coefficient (ICC) was calculated. An average value of 0.857 showed good intra-observer reliability.

## Results

Fifty-one knee CT scans of primary ACL reconstructions were analysed for this study. Mean age of the patients was 30.5±8.4 and 76.7% of them were males. Eight cases (15.6%) had undergone revision. Their measurement data was included only in the revision group, leaving 43 patients in the Primary group. All demographic data is shown on Table I.

**Table I:** Demographic data of study groups.

	Primary cases (n=43)	Revision cases (n=40)	p-value
Age			
Mean ± SD	30.5 ± 8.4	29.4 ± 8.0	0.265*
Median (Min – Max)	28 (19 – 47)	28 (17 – 45)	
Sex			
Male	33 (76.7%)	38 (95.0%)	0.027^+^
Female	10 (23.3%)	2 (5.0%)	
Graft size			
Mean ± SD	8.8 ± 1.0	NA	NA
Median (Min – Max)	8 (8 – 11)		
Time since operation (months)			
Mean ± SD	60.3 ± 15.2		
Median (Min – Max)	63 (22 – 80)		

Notes - * Mann-Whitney U test, ^+^ Chi-Square, NA – not applicable

Of our 8 failed cases, 6 patients had a history of sports trauma and two patients experienced graft failure due to misplacement of the femoral tunnel. Of the revision patients, 5 had been operated with a transtibial technique and had a history of minor trauma, 3 patients had a femoral tunnel misplacement, 1 patient had developed a flexion contracture due to a cyclops formation after re-rupture and 23 patients had a history of sport-related trauma.

Study parameters were compared between the primary cases and the revision ones. The long diameter of the exiting ellipse (5-l) and the distance between the mid-tunnel point and the tibial tuberosity on an axial plane (7) were statistically different between the two groups. Comparative data is shown on Table II.

**Table II:** Study parameters for primary and revision cases.

	**Primary cases (n=43)**	**Revision cases (n=40)**	p-value*
Tunnel height (1) (mm)			
Mean ± SD	30.4 ± 4.5	31.9 ± 6.5	0.734
Median (Min – Max)	29.3 (20.4 – 39.7)	31.5 (22.9 – 50.3)	
Coronal tunnel angle (2) (degree)			
Mean ± SD	18.0 ± 7.1	19.8 ± 4.9	0.643
Median (Min – Max)	18.9 (2.8 – 30.4)	20.5 (9.0 – 27.0)	
Max tunnel width (3) (mm)			
Mean ± SD	10.7 ± 1.6	11.2 ± 2.0	0.527
Median (Min – Max)	10.4 (8.2 – 14.5)	11.0 (7.9 – 15.4)	
Sagittal tunnel inclination (4) (degree)			
Mean ± SD	64.6 ± 8.5	67.3 ± 8.6	0.517
Median (Min – Max)	64.9 (51.2 – 80.7)	68.2 (49.6 – 81.0)	
Long diameter of exit ellipse (5-l) (mm)			
Mean ± SD	11.1 ± 2.4	11.2 ± 2.7	0.011
Median (Min – Max)	10.8 (6.4 – 15.4)	10.9 (7.1 – 16)	
Short diameter of exit ellipse (5-s) (mm)			
Mean ± SD	8.7 ± 2.2	8.5 ± 3.1	0.115
Median (Min – Max)	8.9 (3.1 – 13.2)	8.3 (0.5 – 12.3)	
Axial grid X position (6-x) (%)			
Mean ± SD	46.0 ± 2.9	45.4 ± 2.9	0.141
Median (Min – Max)	45.0 (40.0 – 54.0)	46.0 (36.0 – 49.0)	
Axial grid Y position (6-y) (%)			
Mean ± SD	48.5 ± 7.3	42.4 ± 12.4	0.164
Median (Min – Max)	49.0 (36.0 – 64.0)	49.5 (34.0 – 88.0)	
Axial mid-tunnel distance to TT (7) (mm)			
Mean ± SD	23.6 ± 6.2	25.8 ± 4.9	0.033
Median (Min – Max)	23.9 (11.0 – 37.9)	24.8 (18.6 – 36.0)	
Axial tibial tunnel index (8) (degree)			
Mean ± SD	25.2 ± 12.6	29.0 ± 10.3	0.621
Median (Min – Max)	27.3 (2.3 – 43.0)	29.7 (3.3 – 43.6)	

A wide correlation analysis was performed in-between the study parameters in primary reconstruction cases. Importance was placed on revision and correlating factors. A longer diameter of the intra-articular ellipse (5-l) (r=4.24) and an increased mid-tunnel to TT distance on the axial plane (7) (r=0.414) were significantly correlated with revision. Other parameters significantly correlated to these last two variables were pointed out for a following regression analysis (Table III).

**Table III:** Significant correlation analyses of parameters related to revision of primary case.

	Correlation Coefficient R*	Significance p-value
	**Revision of primary cases**	
Long diameter of exit ellipse (5-l) (mm)	0.424	0.005
Axial mid-tunnel distance to TT (7) (mm)	0.414	0.006
	**Long diameter of exit ellipse**	
Max tunnel width (3) (mm)	0.447	0.003
Axial grid Y position (6-y) (%)	-0.310	0.043
	**Axial tunnel distance to TT**	
Coronal tunnel angle (2) (degree)	0.564	0.000
Axial tibial tunnel index (8) (degree)	0.621	0.000

**Fig. 6:**
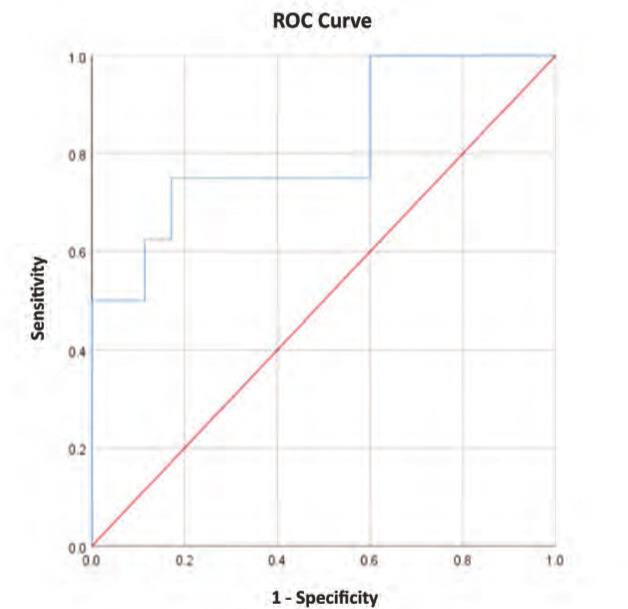
ROC curve analysis for a cut-off value of parameter ‘Axial mid-tunnel distance to TT’.

A univariate logistic regression analysis was performed for all study parameters to investigate a relationship to revision. Significant variables on the univariate analysis were then run through a multivariate regression for adjustment. A multivariate logistic regression analysis showed that an increased distance between the mid-tunnel point and the tibial tuberosity on the axial plane (7) was a significant risk factor for revision (p=0.048) in primary reconstruction cases (Fig. 6).

Finally, an ROC curve analysis to determine a cut-off value for a starting point on the proximal tibia with respect to the tibial tubercle showed that a distance of 27.9mm from the tubercle was an optimal point (75.0% sensitivity and 82.9%% specificity). Data is shown on Table IV.

**Table IV:** Univariate and multivariate logistic regression analysis for revision surgery of primary cases.

**Univariate Logistic regression**			Multivariate Logistic regression analysis		
	HR (95% CI)	p-value		Adjusted HR (95% CI)	p-value
Tunnel height (1)	1.02 (0.86-1.21)	0.797	Tunnel height (1)		
Coronal tunnel angle (2)	1.02 (0.91-1.14)	0.647	Coronal tunnel angle (2)		
Max tunnel width (3)	1.08 (0.69-1.70)	0.708	Max tunnel width (3)		
Sagittal tunnel inclination (4)	1.06 (0.97-1.16)	0.186	Sagittal tunnel inclination (4)		
Long diameter of exit ellipse (5-l)	1.85 (1.15-2.96)	0.011	Long diameter of exit ellipse (5-l)	3.98 (0.81-19.42)	0.088
Short diameter of exit ellipse (5-s)	1.54 (1.01-2.36)	0.052	Short diameter of exit ellipse (5-s)		
Axial grid X position (6-x)	0.69 (0.47-1.03)	0.070	Axial grid X position (6-x)		
Axial grid Y position (6-y)	0.93 (0.83-1.04)	0.246	Axial grid Y position (6-y)		
Axial mid-tunnel distance to TT (7)	1.32 (1.06-1.64)	0.010	Axial mid-tunnel distance to TT (7)	1.65 (1.00 – 2.73)	0.048
Axial tibial tunnel index (8)	1.00 (0.94-1.07)	0.768	Axial tibial tunnel index (8)		

## Discussion

The most important finding of this study is that it identifies a risk factor for revision in primary ACL reconstruction cases. An increased distance between the mid-point of the tibial tunnel at its entrance on the proximal tibia and the middle of the tibial tubercle significantly increases chances of revision. While classical literature advice a tunnel entry just medial to the tibial tuberosity, few studies have thoroughly researched the topic^16^.

Anatomic ACL reconstruction aims to restore the obliquity of the native ligament^3,11,17^. Although improper placement of the tibial tunnels in anterior cruciate ligament reconstruction negatively affects knee kinematics and clinical outcomes, there is still an ongoing debate about what is and is not anatomic^12^. Staubli and Rauschning^18^ suggested placing the tibial tunnels at a distance of 44% of the total midsagittal diameter from the anterior border of the tibia, whereas several cadaveric, radiographic, and 3-dimensional CT studies have evaluated this measure and reported that the centre of the tibial footprint was at an average AP distance of 38.5% to 40.7%^4^.

Another important consideration is the shape and size of the intra-articular aperture. Mehta *et al* found that the anteromedial technique produced a smaller, more circular tibial tunnel and higher peak contact pressure at the tibial tunnel opening under physiologic loading conditions^6^. They concluded that higher peak contact pressure on the graft at the tibial opening may increase graft stress and potentially lead to failure after ACL reconstruction with the anteromedial technique^6^. Contrary to Mehta *et al*^6^, morphometric measurements of this study showed an elongated elliptic shape on the antero-posterior plane being correlated with revision. Although not reaching statistical significance on the multivariate analysis, this parameter has been shown by Sabat *et al*^19^, to lead to a bungee-cord effect and premature graft loosening and revision.

The shape of the exiting aperture is directly related to the distal entry point and its angle. The entry point is usually midway between the posterior cortex of the proximal tibia and the medial edge of the tibial tuberosity^16,20^. Although there is no consensus on the exact region, most surgeons prefer positioning approximately 1cm medial to the tibial tuberosity and anterior to the fibres of the superficial band of the MCL^12^. The axial angle, on the other hand, can be difficult to adjust since the tibial metaphysis may have several anatomic variations in the entry area^21^. Our study shows that a graft entry further than 2.7cm from the tibial tuberosity is a risk factor for revision. Poor long-term clinical outcome has been attributed to inadequate restoration of normal knee kinematics^22^. However, there is a known discrepancy between intra-operative observation and tunnel malalignment as determined by CT scans^12^. Normative data and consensus on this issue are still lacking and treatment should be individualised for all patients.

The strength of early fixation depends on the correct sizing of the tunnel to the size of the available graft. Tunnels that are too large to allow adequate filling by the graft compromise the strength of fixation and healing potential^7,23,24^. L’Insalata *et al*^8^ described the importance of the full graft accommodation at the tunnel opening and how the absence of collagen at the tunnel entrance may allow greater entry of synovial fluid into the bone tunnels. They described this phenomenon as the "windshield wiper" effect. The obliquity of the tunnel in the coronal and sagittal planes has been shown to affect graft accommodation and longevity^20^. Mehta *et al*^6^ called for a less acute angle when drilling the tibial tunnel in the anteromedial technique to reduce the bending angle over the tibial opening and thus exert less stress on the graft. This study found no correlation between tunnel obliquity and revision. Kim *et al*^20^ reported that setting the tibial template at different angles during drilling would affect the severity of intra-articular aperture widening of the tibial tunnel. They suggested that a guide placed at an angle of 40° significantly reduced the severity of aperture widening during ACL reconstruction using the transtibial technique.

ACL reconstruction success rates vary, and graft failure may be due to traumatic or atraumatic causes^7^. Atraumatic causes include technical errors in tunnel placement, failure of fixation, and failure of biological graft incorporation into the bone tunnel^25,26^, all of which may be influenced by aperture and tunnel geometry. Research on this topic has shown that the shape and size of the aperture and tunnel have potential clinical implications. Enlarged tibial tunnel apertures and tunnel misalignment during ACL reconstruction are a major cause of graft failure^7,27^. In addition to primary reconstruction, CT scans of revision cases were evaluated in this study. We observed that revision cases had a significantly more elongated elliptic intra-articular exit point when compared to primary non-revised cases. This parameter was also related to revision of our primary cases but failed to reach statistical significance on a multivariate analysis. On the other hand, the distance between the tibial tuberosity and the mid-point of the tunnel entrance was significantly different between the revision and primary cases, while also remaining so on the final multivariate analysis.

The impact of ACL reconstruction failure in especially young patients is substantial, as they may face the prospect of re-injury, recurrent instability, and long-term joint issues^7,14^. This not only affects their physical health and quality of life but also their professional lives. Young adults often have active lifestyles and careers that require physical agility and stamina, and the loss of working days due to ACL reconstruction failure can result in both financial strain and the disruption of career aspirations. Moreover, the psychological toll of grappling with repeated surgeries and the uncertainty of regaining full functionality can be emotionally taxing for these patients^28^. Therefore, emphasising the precise placement of the femoral and tibial tunnels in ACL reconstruction underscores the importance of meticulous surgical planning, effective post-operative rehabilitation, and injury prevention strategies. These considerations play a pivotal role in reducing the burden on young individuals as they pursue a vibrant, active lifestyle and a successful career.

This study has some limitations that should be noted. Our sample size was small and due to the retrospective study design, no a-priori power analysis was performed. Furthermore, our measurements were made on early postoperative CT scans, and other long-term changes might have eventually affected ultimate revision. We tried to exclude all revisions due to femoral tunnel malposition, hardware failure and slope increase, in order to focus on the tibial tunnel. Despite this, it should be pointed out that the position of the tunnel entry relative to the tibial tubercle cannot, alone, be blamed for the revision surgeries, as revision is often the result of multiple factors. Rather it was found to be an additional risk factor for revision, and it should be interpreted as such. To assess potential short- and long-term risk factors associated with tunnel positioning, early post-operative low-dose CT scans could be considered in further studies. Another limitation is the fact that the measurements made, while statistically significant, are relatively small in number and more clinical research will be needed to evaluate the real impact of these findings on day-to-day clinical practice. Furthermore, the observed optimal distance of 2.7cm could be valid when the surgeon opts to use a 55° drill guide, which is the most commonly used modality. Further research, with in a better organised case-control design, will be required to show is the tunnel entry site distance from the tuberosity changes with the angle of the tibial guide. Additionally, the primary and revision cases were unrelated, which might have introduced bias in the cause-and-effect analyses. Despite this, our study used CT scans for accurate measurements of available patients and the number of CT scans and amount of radiation that would be needed for an in-vivo study with related primary and revision cases is, in our opinion, beyond ethical acceptance. Finally, tunnel widening is common in revision cases. While we cannot assess with surety whether there had been or not subsequent tunnel widening in revision cases, previous studies and clinical experience have shown that tunnel widening tends to happen mostly at its proximal pole or within the metaphysis^29,30^. This study used the distal entry of the tunnel as a measurement reference, a point less frequently affected by the widening phenomenon. Additionally, tunnel widening was not a risk factor in this study, according to our analysis, and therefore we did not emphasise that.

**Table:** 

	**Area Under Curve**	**p-value**	**95% CI**
	.814	.006	0.63 – 0.99
**Cut-off value**	**Sensitivity**	**Specificity**	**Likelihood ratio**
27.9mm	75.0%	82.9%	4.3

## Conclusion

This study shows that a greater distance between the midpoint of the tibial tunnel entrance and the centre of the tibial tubercle is linked to a higher risk of revision. An elongated elliptic shape in the antero-posterior plane also correlates with revision risk. While not statistically significant in multivariate analysis, this factor has been associated with a bungee-cord effect, resulting in premature graft loosening and the need for revision.
